# Ex Vivo Lung Perfusion and Primary Graft Dysfunction Following Lung Transplantation: A Contemporary United Network for Organ Sharing Database Analysis

**DOI:** 10.3390/jcm13154440

**Published:** 2024-07-29

**Authors:** Doug A. Gouchoe, Ervin Y. Cui, Divyaam Satija, Matthew C. Henn, Kukbin Choi, Justin P. Rosenheck, David R. Nunley, Nahush A. Mokadam, Asvin M. Ganapathi, Bryan A. Whitson

**Affiliations:** 1 Division of Cardiac Surgery, Department of Surgery, The Ohio State University Wexner Medical Center, Columbus, OH 43210, USA; doug.gouchoe@osumc.edu (D.A.G.);; 2 COPPER Laboratory, Department of Surgery, The Ohio State University Wexner Medical Center, Columbus, OH 43210, USA; 3 College of Medicine, The Ohio State University, Columbus, OH 43210, USA; 4 Department of Medicine, The Ohio State University Wexner Medical Center, Columbus, OH 43210, USA

**Keywords:** primary graft dysfunction, lung transplant, PGD3, EVLP, ex vivo lung perfusion

## Abstract

**Background:** Primary graft dysfunction (PGD) has detrimental effects on recipients following lung transplantation. Here, we determined the contemporary trends of PGD in a national database, factors associated with the development of PGD grade 3 (PGD3) and ex vivo lung perfusion’s (EVLP) effect on this harmful postoperative complication. **Methods:** The United Network for Organ Sharing database was queried from 2015 to 2023, and recipients were stratified into No-PGD, PGD1/2, or PGD3. The groups were analyzed with comparative statistics, and survival was determined with Kaplan–Meier methods. Multivariable Cox regression was used to determine factors associated with increased mortality. PGD3 recipients were then stratified based on EVLP use prior to transplantation, and a 3:1 propensity match was performed to determine outcomes following transplantation. Finally, logistic regression models based on select criteria were used to determine risk factors associated with the development of PGD3 and mortality within 1 year. **Results:** A total of 21.4% of patients were identified as having PGD3 following lung transplant. Those with PGD3 suffered significantly worse perioperative morbidity, mortality, and had worse long-term survival. PGD3 was also independently associated with increased mortality. Matched EVLP PGD3 recipients had significantly higher use of ECMO postoperatively; however, they did not suffer other significant morbidity or mortality as compared to PGD3 recipients without EVLP use. Importantly, EVLP use prior to transplantation was significantly associated with decreased likelihood of PGD3 development, while having no significant association with early mortality. **Conclusions:** EVLP is associated with decreased PGD3 development, and further optimization of this technology is necessary to expand the donor pool.

## 1. Introduction

Primary graft dysfunction (PGD) is a pattern of acute lung injury that happens early within the postoperative period following lung transplantation, usually within 72 h. Currently, it is graded using the International Society for Heart and Lung Transplantation (ISHLT) definition, which divides PGD into three classifications: Grade 1, pulmonary edema on chest X-ray with PaO_2_/FiO_2_ ratio (P:F) > 300; Grade 2, pulmonary edema on chest X-ray with P:F 200–300; or Grade 3, pulmonary edema on chest X-ray with P:F < 200 or ECMO use (Table 1) [1]. While the reported rates of PGD vary between 20 and 40% [2,3,4], more recently, overall incidence is around 25% [5]. PGD is one of the most common, if not the most important, postoperative complications following lung transplantation, as PGD3 is associated with increased mortality, disability, cost of hospitalization, and chronic allograft dysfunction (CLAD) [1,3,4,5,6,7,8,9]. Perhaps more importantly, compared to lung transplant recipients who do not develop PGD, those who develop PGD lose about a month of life at 1 year, and 5 months at 3 years [5].

Interestingly, the rate of grade 3 PGD (PGD3) continues to rise from around 17% in 2013 [7] to as high as 38.2% in 2022 [5]. Therefore, using a national database, we sought to determine the incidence of PGD in the most recent era (2015–2023) and determine risk factors associated with its development. Additionally, due to the rise in use of ex vivo lung perfusion (EVLP) to expand the donor pool, we sought to determine if EVLP use in those who developed PGD3 altered postoperative outcomes compared to those allografts that did not undergo EVLP.

## 2. Materials and Methods

### 2.1. Study Population

Using the United Network for Organ Sharing (UNOS)/Organ Procurement and Transplant Network (OPTN) Database, adult primary lung transplants from 1 April 2015 to 31 March 2023 were identified. The start date of 1 April 2015 was chosen as that is when the UNOS/OPTN database began capturing data sufficient to calculate PGD at 72 h (PaO_2_, FiO_2_, ECMO requirements) [6]. For the purposes of this study (due to the lack of radiographic information within the UNOS registry and no differentiation of venoarterial or venovenous ECMO), PGD3 was defined as: P:F < 200 or ECMO at 72 h, while Grade 1/2 PGD (PGD1/2) was defined as P:F 200–300, and, finally, no PGD (No-PGD) was defined as: P:F > 300 (Table 1) [6]. For an additional analysis, patients were alternatively grouped as PGD3 and No-PGD3. Recipients without complete data on PaO_2_, FiO_2_, and ECMO at 72 h, as well as recipients with prior lung transplant or multiorgan transplant (to include heart–lung), were excluded. This study was reviewed with institutional review board (IRB) approval (The Ohio State University Wexner Medical Center IRB: #2018H0079).

### 2.2. Statistical Analysis

Continuous variables were assessed for normality and presented as mean ± standard deviation (parametric) or median (interquartile range (IQR)) (nonparametric). Missingness was determined in all variables. All groups were then compared using analysis of variance (continuous parametric), Kruskal–Wallis test (continuous nonparametric), or the chi-square test (categorical). Unadjusted long-term survival was assessed using Kaplan–Meier methods with the log-rank test. Within the study cohort (2015–2023), a multivariable Cox regression model was created. First, a univariate Cox regression was created using recipient age, gender, race, BMI, diabetes, cigarette use, glomerular filtration rate (GFR), medical condition (not hospitalized, hospitalized, in ICU), ventilator use prior to transplant, lung allocation score (LAS), days on waitlist, diagnosis; donor age, race, gender, BMI, history of cigarette use, history of cocaine use, history of alcohol abuse, PaO_2_/FiO_2_ (P:F) ratio, donation after circulatory death (DCD) status, donor cause of death; transplant ischemic time, distance traveled from donor to recipient hospital, laterality, year, and center volume. The final multivariable Cox regression was created using variables which were significant in a univariate model (*p*-value < 0.2), along with our primary exposure variable (PGD grade), to determine independent associations with increased mortality. 

Additionally, previous studies and anecdotal reports have proposed that recipients who develop PGD3 following lung transplantation from allografts that underwent EVLP have improved outcomes, as compared to those who did not undergo EVLP [10]. To further clarify this relationship, patients from 2018 to 2023 who developed PGD3 (at centers which performed EVLP) were stratified into EVLP and No-EVLP, based on EVLP use prior to transplantation. These groups were analyzed with comparative statistics, as described previously. Survival was determined with Kaplan–Meier methods with the log-rank test. Finally, a 3:1 propensity match was performed based on recipient age, sex, BMI, creatinine, diagnosis, LAS, ECMO, and ventilatory use prior to transplant; donor age, sex, race, BMI, history of hypertension, history of cocaine use, history of alcohol abuse, cause of death; and transplant center volume (Supplemental Figure S1). The groups were again compared with the previously mentioned comparative statistics and Kaplan–Meier methods. 

Finally, to determine if EVLP was significantly associated with the development of PGD3, a multivariable logistic regression model using data from the years 2018–2023 was created. First a univariate logistic regression was created using recipient age, gender, race, BMI, diabetes, cigarette use, glomerular filtration rate (GFR), pulmonary artery pressure, medical condition (not hospitalized, hospitalized, in ICU), ventilator use prior to transplant, lung allocation score (LAS), days on waitlist; donor age, race, BMI, gender, history of cigarette use, history of cocaine use, history of alcohol abuse, PaO_2_/FiO_2_ (P:F) ratio, donation after circulatory death (DCD) status, donor cause of death; distance traveled from donor to recipient hospital, laterality, year, and center volume. The final multivariable logistic regression was created using variables which were significant in a univariate model (*p*-value < 0.2), along with our primary exposure variable (EVLP use), to determine independent associations with the development of PGD3. Finally, the same logistic regression criteria and method were used to create a multivariable logistic regression model to determine EVLP’s association with 1-year survival. 

All statistical analyses were performed with R version 3.6.2 (R Core Team, Vienna, Austria) and Microsoft Excel (Redmond, WA, USA). Statistical significance was set at *p* < 0.05 for all analyses.

## 3. Results

A total of 10,342 patients were identified for inclusion into this study. There were 5089 (49.2%) patients in the No-PGD group, 3037 (29.4%) in the PGD1/2 group, and 2216 (21.4%) in the PGD3 group. For recipients who developed PGD3, there was no significant difference in incidence between those received EVLP lungs (19.1%) and those who did not (21.5%) (*p* = 0.21). Regarding the recipients, those in the PGD3 group were significantly older, had a significantly higher body mass index (BMI), had a significantly higher proportion of being Black, and significantly lower glomerular filtration index (GFR) (*p* < 0.05 for all). Additionally, they had a significantly higher lung allocation score (*p* = 0.048). There were significant differences in recipient diagnosis as well. Those in the PGD3 group had significantly higher incidence of restrictive lung disease, while those in the No-PGD group had significantly higher incidences of cystic fibrosis/immunodeficiency and obstructive lung disease (*p* < 0.001). There were no significant differences in hospitalization status prior to transplant or preoperative dialysis, ventilator, or extracorporeal membrane oxygenation (ECMO) use (*p* > 0.05 for all, Table 2). Regarding the donors, those in the PGD3 group were significantly older and had a significantly increased incidence of smoking history and alcohol abuse (*p* < 0.05 for all). Additionally, those in the PGD3 group had significantly lower PaO_2_/FiO_2_ (PF) ratios (*p* < 0.001). There were no significant differences in cause of death (*p* > 0.05) (Table 3).

**Figure 1:**
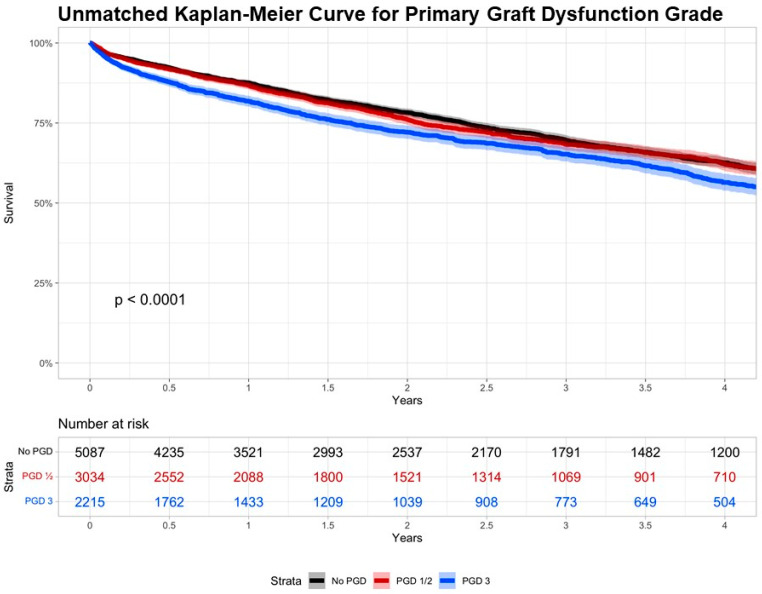
Unmatched Kaplan–Meier curve for primary graft dysfunction grade. Kaplan–Meier survival estimates are plotted; 95% confidence intervals are depicted with shading.

**Figure 2:**
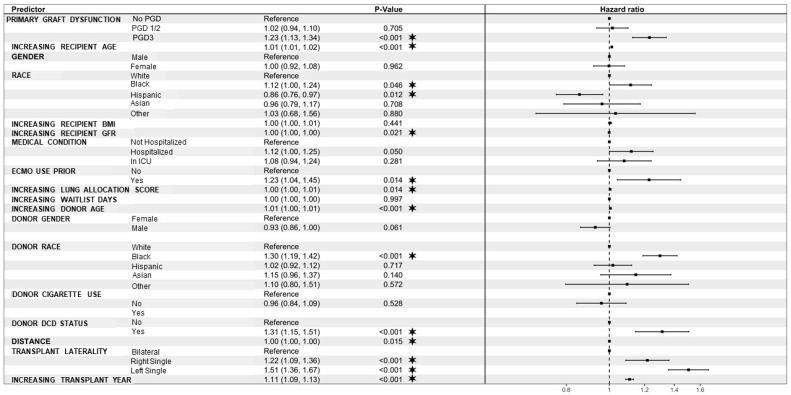
Forest plot of hazard ratios of variables associated with increased mortality following lung transplantation. BMI—body mass index; CVA—cerebrovascular accident; DCD—donation after circulatory death; GSW—gunshot wound; LAS—lung allocation score; PGD—primary graft dysfunction. * denotes statistical significance.

Regarding operative characteristics, those in the PGD3 group had the lowest yearly center volume (*p* < 0.001), significantly longer distance from donor hospital to recipient hospital (*p* < 0.001), and significantly shorter ischemic time (*p* < 0.001). Those in the No-PGD group had significantly higher incidences of bilateral lung transplant (*p* < 0.001). Postoperatively, those in the PGD3 group had significantly longer length of stay (LOS), significantly increased incidence of in-hospital mortality, and significantly increased incidence of postoperative dialysis, airway dehiscence, ECMO use, time on ventilator, and rejection (*p* < 0.05 for all). Cause of death did not differ significantly between groups (*p* > 0.05) (Table 4). When looking at those with PGD3, versus not having PGD3, this relationship held true except that treated rejection was no longer significant between the two groups (*p* = 0.088; Supplemental Table S1). Mid-term survival was significantly lower for those in the PGD3 group (Figure 1): 3-year survival was 69.7% (95% confidence interval (CI): 68.2–71.2%) for the No-PGD group, 68.3% (95% CI: 66.4–70.3%) for the PGD1/2 group, and 65.3% (95% CI: 63.0–67.6%) for the PGD3 group (*p* < 0.0001). Following multivariable Cox regression, PGD3 was independently associated with increased mortality (hazard ratio (HR): 1.23, 95% CI: 1.13–1.34, *p* < 0.001). ECMO use prior to transplant (HR: 1.23, 95% CI: 1.04–1.45, *p* = 0.014) and recipient Black race (HR: 1.12, 95% CI: 1.00–1.24, *p* = 0.046) were also associated with increased mortality. Recipient Hispanic race was associated with decreased mortality (HR: 0.86, 95% CI: 0.76–0.97, *p* = 0.012). Donor Black race (HR: 1.30, 95% CI: 1.19–1.42, *p* < 0.001) and donation after circulatory death (DCD) donors (HR: 1.31, 95% CI: 1.15–1.51, *p* < 0.001) were also associated with increased mortality. Finally, both right-single (HR: 1.22, 95% CI: 1.09–1.36, *p* < 0.001) and left-single (HR: 1.51, 95% CI: 1.36–1.67, *p* < 0.001) transplantation, in addition to increasing transplant year (HR: 1.11, 95% CI: 1.09–1.13, *p* < 0.001), were associated with increased mortality (Figure 2). 

**Figure 3:**
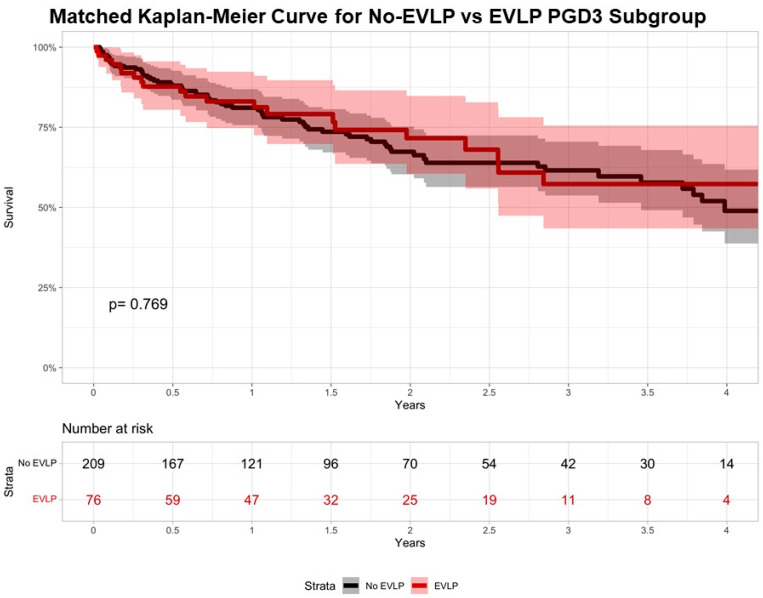
Matched Kaplan–Meier Curve of the recipients with PGD3 who underwent No-EVLP or EVLP. Kaplan–Meier survival estimates are plotted; 95% confidence intervals are depicted with shading.

To further explore EVLP’s relationship with outcomes following the development of PGD3 in the postoperative period, patients whose transplants were performed at centers that use EVLP allografts were queried from 2018 to 2023, and they were then into stratified EVLP and No-EVLP. There were 677 (89.1%) No-EVLP patients and 82 (10.9%) EVLP patients included in this subgroup analysis. Unmatched recipients in the No-EVLP had significantly higher LAS (*p* = 0.002) and were more often in the ICU (*p* = 0.035; Supplemental Table S2). Unmatched donors in the EVLP group had significantly lower PF ratios (*p* = 0.003), significantly higher BMI (*p* = 0.001), and were more often from DCD donors (*p* < 0.001; Supplemental Table S3). Regarding unmatched postoperative outcomes, those in the EVLP group had significantly longer ischemic times, distance from donor to recipient hospital, and significantly increased incidences of postoperative ECMO, and treated rejection in the first year (*p* < 0.05 for all, Supplemental Table S4). Unmatched 3-year survival was 65.3% (95% CI: 60.8–70.0%) for the No-EVLP group and 54.2% (95% CI: 40.9–71.6%) for the EVLP group (*p* = 0.17, Supplemental Figure S2). Following propensity matching, two well-matched groups were created (Supplemental Figure S1; Supplemental Tables S5 and S6), with 209 in the No-EVLP group and 76 in the EVLP group. Postoperatively, the matched EVLP group had significantly longer ischemic times, distance from donor to recipient hospital, and significantly increased incidences of postoperative ECMO use (*p* < 0.05 for all, Table 5). Matched 3-year survival was 61.5% (95% CI: 53.7–70.5%) for the No-EVLP group and 57.3% (95% CI: 43.4–75.5%) for the EVLP group (*p* = 0.769, Figure 3). 

Following logistic regression for development of PGD3, EVLP use was significantly associated with decreased likelihood of developing PGD3 (odds ratio (OR): 0.70, 95% CI: 0.57–0.86, *p* < 0.001). Black, Hispanic, and Asian donor race were all associated with decreased likelihood of developing PGD3 as well (*p* < 0.05 for all). Additionally, right-single (OR: 1.50, 95% CI: 1.25–1.79, *p* < 0.001) and left-single (OR: 1.39, 95% CI: 1.17–1.65, *p* < 0.001) transplant were both significantly associated with the development of PGD3 (Table 6). Logistic regression for mortality within 1 year revealed that EVLP (OR: 1.16, 95% CI: 0.95–1.43, *p* = 0.15) and PGD3 (OR: 1.13, 95% CI: 0.99–1.28, *p* = 0.07) were not significantly associated with death. Recipient ECMO use prior to transplant, hospitalization status, donor Black race, and DCD donors were significantly associated with mortality within 1 year (*p* < 0.05 for all). Interestingly, right-single lung transplant was protective against mortality within 1 year (OR: 0.83, 95% CI: 0.69–0.99, *p* = 0.04) (Table 7). 

## 4. Discussion

Here, we reinforced PGD’s detrimental effects on postoperative outcomes, as well as long-term survival, using a national database [3,4,5,6]. Furthermore, we again demonstrated PGD3’s independent association with long-term mortality [3,4]. Here, we detailed national rates of PGD3 to be 21.4% during our study period within the UNOS database. This is consistent with the most recent work carried out by the Lung Transplant Outcomes Group (LTOG), which found that 25.7% of recipients within their database suffered from PGD3 postoperatively [5]. It is to be noted that our definition of PGD1/2 is limited by the UNOS database, namely, due to the lack of chest X-ray data [1,6]; however, the overall incidence of PGD within our cohort (49.2%) is somewhat similar to the LTOG’s findings (43%) [5]. While the incidence of any PGD within the UNOS database is perhaps an overestimation, the incidence of PGD3 more closely resembles current trends within granular databases. Therefore, using this definition of PGD3 within the UNOS database (P:F < 200 or ECMO at 72 h) is an appropriate surrogate that will allow other transplant providers to more closely study PGD3 and its effects within a large national database. 

Additionally, we sought to determine EVLP’s association with the development of PGD3, and postoperative outcomes and survival once PGD3 had developed. Previously, large-volume centers have reported that EVLP allografts have improved outcomes when the recipient develops PGD3 as compared to those with non-EVLP allografts [10]. Benazzo et al. reported that these EVLP allografts that develop PGD3 at 72 h had a nonsignificant decreased ventilatory support time (6 days), LOS (40 days), and mortality at 90 days (8%) as compared to non-EVLP allografts that developed PGD3 at 72 h [10]. Within our matched cohort, those who developed PGD3 after the use of EVLP only suffered worse perioperative morbidity in terms of ECMO use following transplantation. Notably, our EVLP cohort had an LOS of 28.5 days, and the majority (59.5%) spent 5+ days on the ventilator. Mortality was 8.1% at 90 days, which was also similar to that previously described [10]. Finally, similarly to Benazzo et al., the PGD3 rate at 72 h in our EVLP cohort was 19.1%, compared to 19.0% within their cohort [10]. Ultimately, our EVLP cohort showed similar perioperative outcomes within our matched cohort, as well as survival. Therefore, the use of EVLP prior to the development of PGD3 does not offer any advantages in terms of mitigating PGD3-associated postoperative morbidity, as once thought. 

However, our study importantly found that EVLP use was significantly associated with a decreased likelihood of developing PGD3. Though the rates of PGD3 between the groups were not significantly different, this association was still significant after adjusting for a variety of factors within our logistic regression model. Furthermore, EVLP use was not significantly associated with mortality within 1 year. While it has been reported previously that PGD3 rates are lower in EVLP allograft recipients [11], this association has not always held true [12]. However, there have been no direct reports delineating the decreased likelihood of developing PGD3 in EVLP allograft recipients. The reasons behind this could be severalfold. Of importance, ischemia reperfusion injury (IRI) is the underlying mechanism for PGD development during the transplantation process [13,14]. Though the EVLP group had significantly longer total ischemic times, EVLP does suspend ischemic time and restore circulation and nutritional delivery to lung tissues [15]. However, the EVLP protocols used are not uniform and could consist of two cold ischemic periods, or none [16]. Therefore, it is unlikely that decreasing nonperfusion ischemic time alone would explain the decreased likelihood of PGD3. Recent transcriptomic studies have shown that EVLP use modifies gene expression of circulating leukocytes [17], decreases activation of the innate immune system [18], and upregulates vascular functions [19]. Therefore, EVLP use, in itself, due to perfusate composition, perfusion, protocol, and center-level expertise, contributes to the decreased likelihood of PGD3. However, due to the lack of granular data, further center-specific (and protocol-specific) research is needed to substantiate these findings. EVLP is a valuable tool, and its future applications in terms of actively repairing prospective allografts are exciting. In this current analysis, the use of EVLP decreased the likelihood of PGD3, and had no independent association with mortality. Further research is imperative to optimize this technology and further progress its benefits in order to promote organ stewardship and further expand the donor pool.

Transplant laterality has several important associations to highlight as well. Within the No-PGD group, there was a significantly higher proportion of bilateral lung transplants. Additionally, right-single and left-single transplant were independently associated with increased likelihood of PGD3 and long-term mortality, while interesting right-single lung transplantation was protective against mortality within the first year following transplant. While bilateral lung transplantation is well known to confer long-term survival advantages [20,21] and decrease the incidence of PGD3 [7], it is interesting to see that right-single conferred a 1-year survival advantage given the paradoxical association with increased PGD3. This is most likely due to the patient population that would only qualify for right-single lung transplantation, i.e., isolated right-sided disease, or inability to offer bilateral lung transplantation. While this population might have a survival advantage due to a less morbid procedure, their underlying pathophysiology is unlikely to be corrected; thus, increased long-term mortality is still prevalent within this population [22]. Though PGD3 is known to be mitigated by bilateral lung transplantation [7], exact mechanisms are still not elucidated. A possibility is that during bilateral lung transplantation, the new healthy opposing lung can better handle the variation in cardiac output better than the single diseased lung, which would account for better adaptation to vascular hydrostatic pressure, decreasing pulmonary edema and eventual PGD. Though this analysis cannot account for mechanical circulatory support during transplantation, there are several ongoing investigations examining the routine use of ECMO during bilateral lung transplantation. This could offer another explanation for this phenomena, as ECMO use allows for controlled reperfusion and off-loading of cardiac output, which, again, would place less strain on pulmonary vasculature, decrease edema, and PGD [23]. 

Another important finding to highlight as well was the intersection of race, PGD3, and mortality. While recipient Black race has been noted to be associated with increased PGD3 risk [24], it is interesting to note that Black donors had the opposite effect, in addition to other minority groups, as compared to White donors. This finding is noteworthy, as our study, in addition to others, notes that Black donors are associated with increased mortality and worse post-transplant outcomes following lung transplantation [25,26]. While there are no current explanations that would account for this difference, it is possible that due to the overall low usage of minority donor organs [27], this observation is a result of being underpowered or due to comorbidity confounders where granularity on disease burden is not available. Therefore, further research is needed to determine the association of donor race and perioperative morbidity. Finally, as DCD allograft use is increasing within the United States, it is interesting to note its independent association with increased mortality within our analysis. Previous studies have reported no differences in survival with the use of DCD allografts [28,29]. Although in large-database studies DCD allografts have been shown to have increased perioperative complications, short-term survival was not impacted unless used in combination with EVLP [30]. However, some multicenter studies have also shown that there is similar survival with DCD EVLP allografts, as compared to donation after brain death donor allografts, with and without EVLP use prior to transplantation [12]. While this study did not look to specifically delve into the topic of DCD allograft use, their use was, nonetheless, significantly associated with increased mortality in our multivariable analyses. Ultimately, more large-database studies, preferably multicenter, are needed to clarify this relationship of DCD allograft use. 

### Limitations

There are some limitations to this study worth considering. First, the UNOS database is a retrospective administrative database, does not include granular data (to include treatment of PGD), and is, furthermore, subject to information bias. As previously mentioned, the UNOS database does not include chest X-ray data, and does have missingness in terms of PaO_2_ and FiO_2_ data; therefore, we cannot exclude a certain degree of selection bias that influenced the rate of PGD observed in the analysis, especially PGD1/2. Our definition of PGD3 may have underestimated the true incidence of PGD within our cohort according to current work as well [5]. Additionally, we are unable to use the true ISHLT definition in our study, which affects our results as well [1]. Data regarding long-term morbidity, such as dialysis, rejection, functional status, and freedom from oxygen utilization, were unavailable for analysis, which prevented further analysis of quality of life. 

## 5. Conclusions

PGD’s detrimental effects on postoperative morbidity, mortality, and long-term survival are again reinforced in this current analysis. Within our study, we detail national rates of PGD3 to be 21.4% over our study period, which is consistent with the most recent work carried out by the LTOG [5]. While the incidence of any PGD within the UNOS database is perhaps an overestimation, the incidence of PGD3 more closely resembles current trends within granular databases. Therefore, using this definition of PGD3 within the UNOS database (P:F < 200 or ECMO at 72 h) is an appropriate surrogate that will allow other transplant providers to more closely study PGD3 and its effects within a large national database. 

Though EVLP use prior to the development does not offer any advantages in terms of mitigating PGD3’s detrimental effects in the postoperative period, we importantly found that EVLP use was significantly associated with a decreased likelihood of developing PGD3. EVLP is an important tool in a lung transplant provider’s armament, and future studies should focus on further optimizing this technology in order to actively repair donor organs to expand the donor pool. 

## Figures and Tables

**Table 1:** Primary graft dysfunction definitions.

	ISHLT Definition	Study Definition
No-PGD	No pulmonary edema on chest X-ray with P:F > 300	P:F > 300
Grade 1	Pulmonary edema on chest X-ray with P:F > 300	P:F 200–300
Grade 2	Pulmonary edema on chest X-ray with P:F 200–300	P:F 200–300
Grade 3	Pulmonary edema on chest X-ray with P:F < 200 or ECMO use	P:F < 200 or ECMO at 72 h

ECMO, extracorporeal membrane oxygenation; ISHLT, International Society for Heart-Lung Transplantation; P:F, PaO_2_/FiO_2_ ratio.

**Table 2:** Recipient characteristics 2015–2023.

Variable	Overall (n = 10,342)	No-PGD (n = 5089)	PGD1/2 (n = 3037)	PGD3 (n = 2216)	*p*-Value
Age	61 (53, 66)	61 (52, 66)	61 (53, 66)	61 (53, 67)	0.002
Male sex	6079 (58.8%)	2978 (58.5%)	1796 (59.1%)	1305 (58.9%)	0.854
Race					0.035
White	7439 (71.9%)	3722 (73.1%)	2161 (71.2%)	1556 (70.2%)	
Black	1171 (11.3%)	545 (10.7%)	343 (11.3%)	283 (12.8%)	
Other	1732 (16.7%)	822 (16.2%)	533 (17.6%)	377 (17%)	
BMI (kg/m^2^)	26.3 (22.8, 29.4)	25.6 (22, 28.8)	26.8 (23.4, 29.6)	27.3 (23.8, 30.1)	<0.001
Diabetes	2072 (20.1%)	989 (19.5%)	617 (20.3%)	466 (21%)	0.271
Former smoker >20 pack years	5505 (53.2%)	2672 (52.5%)	1633 (53.8%)	1200 (54.2%)	0.33
GFR (mL/min/1.73 m^2^)	92 (72.2, 120.9)	93.5 (73.5, 124.3)	90.8 (71.4, 118.7)	89.8 (70.7, 115.9)	<0.001
Preoperative dialysis	52 (13.1%)	24 (11.8%)	10 (9.3%)	18 (20.7%)	0.048
mPAP (mm Hg)	26 (21, 33)	26 (21, 33)	26 (20.1, 33)	26 (21, 34)	0.284
Diagnosis					<0.001
Cystic fibrosis/immunodeficiency	634 (6.1%)	411 (8.1%)	135 (4.4%)	88 (4%)	
Obstructive lung disease	2100 (20.3%)	1193 (23.4%)	549 (18.1%)	358 (16.2%)	
Pulmonary vascular disease	691 (6.7%)	300 (5.9%)	202 (6.7%)	189 (8.5%)	
Restrictive lung disease	6917 (66.9%)	3185 (62.6%)	2151 (70.8%)	1581 (71.3%)	
LAS	43.3 (36.5, 62.3)	43.1 (36.1, 62.8)	43.4 (36.7, 59.9)	43.5 (37.1, 65.2)	0.032
Hospitalized prior to transplant					0.074
Not hospitalized	7308 (70.7%)	3596 (70.7%)	2175 (71.6%)	1537 (69.4%)	
Hospitalized	1184 (11.5%)	608 (11.9%)	334 (11%)	242 (10.9%)	
In ICU	1848 (17.9%)	884 (17.4%)	527 (17.4%)	437 (19.7%)	
Preoperative ventilator	814 (7.9%)	397 (7.8%)	226 (7.4%)	191 (8.6%)	0.284
Preoperative ECMO	978 (9.5%)	478 (9.4%)	288 (9.5%)	212 (9.6%)	0.971
Days on wait list	42 (13, 130)	42 (13, 137)	43 (13, 128)	40 (13, 118)	0.221

Variables are either presents as median, interquartile range or n, percentage when appropriate. BMI—body mass index; ECMO—extracorporeal membrane oxygenation; GFR—glomerular filtration rate; ICU—intensive care unit; LAS—lung allocation score; mPAP—mean pulmonary artery pressure.

**Table 3:** Donor characteristics 2015–2023.

Variable	Overall (n = 10,470)	No-PGD (n = 5140)	PGD1/2 (n = 3080)	PGD3 (n = 2250)	*p*-Value
Age	35 (25, 47)	34 (24, 47)	35 (25, 48)	36 (26, 48)	<0.001
Male sex	6073 (58.7%)	2993 (58.8%)	1798 (59.2%)	1282 (57.9%)	0.607
Coronary artery disease	718 (7%)	337 (6.6%)	221 (7.3%)	160 (7.2%)	0.442
Smoking history	777 (7.7%)	326 (6.5%)	254 (8.5%)	197 (9.1%)	<0.001
Recent cocaine use	2053 (20.2%)	970 (19.4%)	617 (20.8%)	466 (21.4%)	0.099
Diabetes	898 (8.8%)	461 (9.1%)	248 (8.3%)	189 (8.6%)	0.376
Hypertension	2614 (25.5%)	1306 (25.9%)	761 (25.4%)	547 (24.9%)	0.677
Alcohol abuse	1785 (17.7%)	816 (16.5%)	543 (18.4%)	426 (19.7%)	0.002
BMI (kg/m^2^)	25.7 (22.6, 29.5)	25.7 (22.6, 29.5)	25.9 (22.8, 29.5)	25.6 (22.5, 29.4)	0.104
PF ratio	438 (379, 492)	445 (388, 496)	434.5 (375, 486)	427 (367, 490)	<0.001
Donor cause of death					0.681
Neuro (seizure/CVA)	3085 (29.8%)	1497 (29.4%)	922 (30.4%)	666 (30.1%)	
Drug overdose	1523 (14.7%)	765 (15%)	436 (14.4%)	322 (14.5%)	
Asphyxiation	554 (5.4%)	267 (5.2%)	175 (5.8%)	112 (5.1%)	
Cardiovascular	949 (9.2%)	472 (9.3%)	280 (9.2%)	197 (8.9%)	
Trauma (GSW/stab/blunt)	3859 (37.3%)	1891 (37.2%)	1121 (36.9%)	847 (38.2%)	
Drowning	26 (0.3%)	18 (0.4%)	6 (0.2%)	2 (0.1%)	
Other	344 (3.3%)	178 (3.5%)	96 (3.2%)	70 (3.2%)	
DCD	685 (6.6%)	334 (6.6%)	207 (6.8%)	144 (6.5%)	0.875

Variables are either presents as median, interquartile range, or n; percentage when appropriate. BMI—body mass index; CAD—coronary artery disease; CVA—cerebral vascular accident; DCD—donation after circulatory death; GSW—gunshot wound; PF ratio—PaO2/FiO_2_ ratio.

**Table 4:** Operative and postoperative characteristics 2015–2023.

Variable	Overall (n = 10,470)	No-PGD (n = 5140)	PGD1/2 (n = 3080)	PGD3 (n = 2250)	*p*-Value
Center volume yearly	26.9 (14.9, 40.7)	32.1 (15.5, 41)	24.7 (14.9, 40.7)	22.8 (13.7, 34.4)	<0.001
Bilateral lung transplant	8304 (80.3%)	4245 (83.4%)	2340 (77%)	1719 (77.6%)	<0.001
Distance traveled	163 (45, 311)	162 (45, 304)	158 (44, 302)	173 (51.8, 351)	0.017
Ischemic time	5.6 (4.6, 6.8)	5.6 (4.6, 6.9)	5.6 (4.5, 6.7)	5.5 (4.5, 6.6)	<0.001
Length of stay (days)	23 (15, 41)	21 (14, 36)	24 (15, 41)	27 (17, 51)	<0.001
In-hospital mortality	607 (6%)	242 (4.9%)	168 (5.7%)	197 (9.2%)	<0.001
Postoperative dialysis	1260 (12.2%)	512 (10.1%)	362 (11.9%)	386 (17.4%)	<0.001
Postoperative stroke	338 (3.3%)	178 (3.5%)	83 (2.7%)	77 (3.5%)	0.14
Airway dehiscence	221 (2.1%)	95 (1.9%)	63 (2.1%)	63 (2.9%)	0.029
Postoperative ECMO	1377 (13.3%)	626 (12.3%)	345 (11.4%)	406 (18.3%)	<0.001
Postoperative ventilator					<0.001
<2 days	4037 (39.4%)	2286 (45.4%)	1112 (36.9%)	639 (29.1%)	
2–5 days	2307 (22.5%)	1129 (22.4%)	692 (23%)	486 (22.1%)	
5+ days	3757 (36.7%)	1557 (30.9%)	1164 (38.6%)	1036 (47.2%)	
None	140 (1.4%)	62 (1.2%)	44 (1.5%)	34 (1.5%)	
Acute rejection (hospitalization)					<0.001
Yes and treated with immunosuppressant	777 (7.5%)	322 (6.3%)	244 (8%)	211 (9.5%)	
Yes and not treated with immunosuppressant	127 (1.2%)	56 (1.1%)	35 (1.2%)	36 (1.6%)	
No	9435 (91.3%)	4710 (92.6%)	2757 (90.8%)	1968 (88.8%)	
Treated rejection (1st year)	1512 (19.8%)	709 (18.7%)	472 (20.6%)	331 (21.4%)	0.041
Cause of death					0.102
Graft failure	516 (16.4%)	237 (16.3%)	145 (15.8%)	134 (17.3%)	
Malignancy	200 (6.4%)	103 (7.1%)	57 (6.2%)	40 (5.2%)	
Cardio/cerebrovascular	415 (13.2%)	188 (13%)	125 (13.7%)	102 (13.2%)	
Pulmonary	647 (20.6%)	289 (19.9%)	199 (21.7%)	159 (20.6%)	
Infection	758 (24.1%)	339 (23.4%)	244 (26.7%)	175 (22.6%)	
Other	603 (19.2%)	295 (20.3%)	145 (15.8%)	163 (21.1%)	

Variables are either presents as median, interquartile range, or n; percentage when appropriate. ECMO—extracorporeal membrane oxygenation.

**Table 5:** Matched operative and postoperative characteristics of No-EVLP vs. EVLP subgroup who developed PGD3 2018–2023.

Variable	Overall (n = 285)	No EVLP (n = 209)	EVLP (n = 76)	*p*-Value
Center volume yearly	16.7 (11.5, 23.3)	16.7 (12.3, 23.1)	16.5 (9.1, 31.1)	0.561
Bilateral lung transplant	244 (85.6%)	179 (85.6%)	65 (85.5%)	0.707
Distance traveled	180 (94, 428)	157 (76, 314)	312 (145, 536.8)	<0.001
Ischemic time	6.4 (5, 8.7)	5.8 (4.7, 6.9)	12 (8.3, 15.3)	<0.001
Length of stay (days)	35 (18, 62)	36 (18, 64)	29 (18, 47.8)	0.29
In-hospital mortality	23 (8.4%)	16 (8%)	7 (9.5%)	0.887
Postop dialysis	65 (22.8%)	47 (22.5%)	18 (23.7%)	0.958
Postop stroke	11 (3.9%)	9 (4.3%)	2 (2.6%)	0.758
Airway dehiscence	5 (1.8%)	4 (1.9%)	1 (1.3%)	0.999
Postoperative ECMO	70 (24.6%)	41 (19.6%)	29 (38.2%)	0.002
Postoperative ventilator				0.869
<2 Days	62 (22.1%)	48 (23.3%)	14 (18.9%)	
2–5 Days	55 (19.6%)	39 (18.9%)	16 (21.6%)	
5+ Days	155 (55.4%)	113 (54.9%)	42 (56.8%)	
None	8 (2.9%)	6 (2.9%)	2 (2.7%)	
Acute rejection (hospitalization)				0.369
Yes and treated with immunosuppressant	32 (11.2%)	22 (10.5%)	10 (13.2%)	
Yes and not treated with immunosuppressant	9 (3.2%)	5 (2.4%)	4 (5.3%)	
No	244 (85.6%)	182 (87.1%)	62 (81.6%)	
Treated rejection (1st year)	42 (21.4%)	27 (18.8%)	15 (28.8%)	0.186
Cause of death				0.694
Graft failure	15 (17.2%)	12 (18.2%)	3 (14.3%)	
Malignancy	2 (2.3%)	2 (3%)	0 (0%)	
Cardio/cerebrovascular	11 (12.6%)	8 (12.1%)	3 (14.3%)	
Pulmonary	25 (28.7%)	21 (31.8%)	4 (19%)	
Infection	24 (27.6%)	16 (24.2%)	8 (38.1%)	
Other	10 (11.5%)	7 (10.6%)	3 (14.3%)	
Perfused by				
Organ procurement organization			2 (2.7%)	
Transplant program			49 (65.3%)	
External perfusion center			24 (32%)	
Perfusion time (minutes)			254.5 (219.8, 345.8)	

Variables are either presents as median, interquartile range, or n; percentage when appropriate. ECMO—extracorporeal membrane oxygenation.

**Table 6:** Logistic regression for development of primary graft dysfunction Grade 3 from 2018 to 2023.

Variable	Odds Ratio	Lower Bound	Upper Bound	*p*-Value
EVLP use				
No	Reference			
Yes	0.70	0.57	0.86	<0.001
Recipient age	1.00	0.99	1.00	0.81
Recipient BMI	1.06	1.05	1.07	<0.001
Recipient glomerular filtration rate	1.00	1.00	1.00	0.49
Recipient medical condition				
Not hospitalized	Reference			
Hospitalized	0.91	0.77	1.08	0.28
In intensive care unit	1.05	0.88	1.24	0.61
Lung allocation score	1.00	1.00	1.01	0.08
Recipient days on waitlist	1.00	1.00	1.00	0.37
Donor age	1.01	1.00	1.01	0.001
Donor race				
White	Reference			
Black	0.67	0.59	0.77	<0.001
Hispanic	0.79	0.69	0.89	<0.001
Asian	0.69	0.53	0.91	0.008
Other	0.67	0.42	1.07	0.09
Donor cigarette use				
No	Reference			
Yes	1.16	0.96	1.39	0.12
Donor PaO_2_/FiO_2_ ratio	1.00	1.00	1.00	0.15
Transplant laterality				
Bilateral	Reference			
Right-single	1.50	1.25	1.79	<0.001
Left-single	1.39	1.17	1.65	<0.001
Transplant year	0.97	0.94	1.01	0.11
Yearly center volume	1.00	1.00	1.00	<0.001

BMI, body mass index; EVLP, ex vivo lung perfusion. Continuous variables listed represent increasing value associated with the development of primary graft dysfunction Grade 3.

**Table 7:** Logistic regression for mortality within 1 year from 2018 to 2023.

Variable	Odds Ratio	Lower Bound	Upper Bound	*p*-Value
EVLP use				
No	Reference			
Yes	1.16	0.95	1.43	0.15
Primary graft dysfunction				
No-PGD	Reference			
PGD 1/2	0.99	0.88	1.11	0.89
PGD 3	1.13	0.99	1.28	0.07
Recipient age	1.01	1.01	1.02	<0.001
Recipient BMI	1.01	1.00	1.02	0.06
Recipient smoking history				
No	Reference			
Yes	0.86	0.78	0.96	0.01
Recipient medical condition				
Not hospitalized	Reference			
Hospitalized	1.26	1.07	1.48	0.01
In intensive care unit	1.11	0.92	1.35	0.28
ECMO use prior				
No	Reference			
Yes	1.26	1.01	1.58	0.04
Lung allocation score	1.00	1.00	1.01	0.50
Recipient days on waitlist	1.00	1.00	1.00	0.02
Donor age	1.01	1.00	1.01	<0.001
Donor race				
White	Reference			
Black	1.30	1.14	1.49	<0.001
Hispanic	1.06	0.93	1.21	0.37
Asian	1.25	0.95	1.64	0.11
Other	1.11	0.70	1.78	0.65
Donation after circulatory death donor				
No	Reference			
Yes	1.35	1.12	1.63	<0.001
Donor PaO_2_/FiO_2_ ratio	1.00	1.00	1.00	0.02
Donor cocaine use				
No	Reference			
Yes	1.11	0.98	1.25	0.09
Transplant laterality				
Bilateral	Reference			
Right-single	0.83	0.69	0.99	0.04
Left-single	1.04	0.87	1.23	0.68
Distance	1.00	1.00	1.00	<0.001

BMI, body mass index; EVLP, ex vivo lung perfusion. Continuous variables listed represent increasing value associated with mortality within 1 year.

## Data Availability

Restrictions apply to the availability of these data. Data were obtained from the United Network for Organ Sharing (UNOS) and are available at https://unos.org/data/ with the permission of them.

## Supplementary Materials

The following supporting information can be downloaded at: https://www.mdpi.com/article/10.3390/jcm13154440/s1. Table S1. Operative and postoperative characteristics of PGD3 vs. No-PGD3, 2015–2023; Table S2. Unmatched recipient characteristics of No-EVLP vs. EVLP subgroup who developed PGD3 (2018–2023); Table S3. Unmatched donor characteristics of No-EVLP vs. EVLP subgroup who developed PGD3 2018–2023; Table S4. Unmatched operative and postoperative characteristics of No-EVLP vs. EVLP subgroup who developed PGD3 2018–2023; Table S5. Matched recipient characteristics of No-EVLP vs. EVLP subgroup who developed PGD3 2018–2023; Table S6. Matched donor characteristics of No-EVLP vs. EVLP subgroup who developed PGD3 2018–2023; Figure S1: Love plot of matched and unmatched standard mean deviations for No-EVLP vs. EVLP PGD3 subgroup. Figure S2: Unmatched Kaplan–Meier curve of the recipients with PGD3 who underwent No-EVLP or EVLP. Kaplan–Meier survival estimates are plotted; 95% confidence intervals are depicted with shading.
