# Supplementary material for: Ex Vivo Lung Perfusion and Primary Graft Dysfunction Following Lung Transplantation: A Contemporary United Network for Organ Sharing Database Analysis
Source: J Clin Med. 2024 Jul 29;13(15):4440. doi: 10.3390/jcm13154440 (PMC11313603; doi:10.3390/jcm13154440)
Supplement: Supplementary file 1 [file jcm-13-04440-s001.zip › jcm-3083463-supplementary.pdf]

SUPPLEMENTAL TABLES

Supplemental Table S1. Operative and postoperative characteristics of PGD3 vs. No-PGD3, 2015–2023.

Variable	Overall (n = 10,342)	No-PGD3 (n = 8,126)	PGD3 (n = 2,216)	P-Value
Center volume yearly	26.9 (14.9, 40.7)	32.1 (14.9, 40.7)	22.8 (13.7, 34.4)	< 0.001
Bilateral lung transplant	8,304 (80.3%)	6,585 (81%)	1,719 (77.6%)	< 0.001
Distance traveled	163 (45, 311)	160 (44.2, 304)	173 (51.8, 351)	0.005
Ischemic time	5.6 (4.6, 6.8)	5.6 (4.6, 6.8)	5.5 (4.5, 6.6)	0.001
Length of stay (days)	23 (15, 41)	22 (15, 38)	27 (17, 51)	< 0.001
In-hospital mortality	607 (6%)	410 (5.2%)	197 (9.2%)	< 0.001
Postoperative dialysis	1,260 (12.2%)	874 (10.8%)	386 (17.4%)	< 0.001
Postoperative stroke	338 (3.3%)	261 (3.2%)	77 (3.5%)	0.565
Airway dehiscence	221 (2.1%)	158 (2%)	63 (2.9%)	0.012
Postoperative ECMO	1,377 (13.3%)	971 (11.9%)	406 (18.3%)	< 0.001
Postoperative ventilator				< 0.001
<2 days	4,037 (39.4%)	3,398 (42.2%)	639 (29.1%)	
2–5 days	2,307 (22.5%)	1,821 (22.6%)	486 (22.1%)	
5+ days	3,757 (36.7%)	2,721 (33.8%)	1,036 (47.2%)	
None	140 (1.4%)	106 (1.3%)	34 (1.5%)	
Acute rejection (hospitalization)				< 0.001
Yes and treated with immunosuppressant	777 (7.5%)	566 (7%)	211 (9.5%)	
Yes and not treated with immunosuppressant	127 (1.2%)	91 (1.1%)	36 (1.6%)	
No	9,435 (91.3%)	7,467 (91.9%)	1,968 (88.8%)	
Treated rejection (1st year)	1,512 (19.8%)	1,181 (19.4%)	331 (21.4%)	0.088
Cause of death				0.341
Graft failure	516 (16.4%)	382 (16.1%)	134 (17.3%)	
Malignancy	200 (6.4%)	160 (6.8%)	40 (5.2%)	
Cardio/cerebrovascular	415 (13.2%)	313 (13.2%)	102 (13.2%)	
Pulmonary	647 (20.6%)	488 (20.6%)	159 (20.6%)	
Infection	758 (24.1%)	583 (24.6%)	175 (22.6%)	
Other	603 (19.2%)	440 (18.6%)	163 (21.1%)	

Variables are either presented as median, interquartile range, or n; percentage when appropriate. ECMO: extracorporeal membrane oxygenation.

Supplemental Table S2. Unmatched recipient characteristics of No-EVLP vs. EVLP subgroup who developed PGD3 (2018–2023).

Variable	Overall (n = 759)	No-EVLP (n = 677)	EVLP (n = 82)	P-Value
Age	61 (53, 67)	61 (53, 67)	60.5 (54.2, 66)	0.876
Male sex	440 (58%)	393 (58.1%)	47 (57.3%)	0.993
Race				0.221
White	529 (69.7%)	468 (69.1%)	61 (74.4%)	
Black	95 (12.5%)	83 (12.3%)	12 (14.6%)	
Other	135 (17.8%)	126 (18.6%)	9 (11%)	
BMI (kg/m2)	27.03 +/- 4.17	26.95 +/- 4.25	27.69 +/- 3.45	0.127
Diabetes	148 (19.5%)	137 (20.2%)	11 (13.4%)	0.185
Former smoker >20 pack years	403 (53.1%)	360 (53.2%)	43 (52.4%)	0.993
GFR (ml/min/1.73 m2)	91.6 (71.9, 118)	91.6 (72.3, 118.5)	91.7 (69.9, 116.6)	0.855
Preoperative dialysis	5 (0.66%)	5 (0.74%)	0 (0%)	0.999
mPAP (mm Hg)	26 (21, 35)	26 (21, 35)	25 (19, 31)	0.085
Diagnosis				0.272
Cystic fibrosis/immunodeficiency	18 (2.4%)	17 (2.5%)	1 (1.2%)	
Obstructive lung disease	110 (14.5%)	93 (13.7%)	17 (20.7%)	
Pulmonary vascular disease	57 (7.5%)	53 (7.8%)	4 (4.9%)	
Restrictive lung disease	574 (75.6%)	514 (75.9%)	60 (73.2%)	
LAS	44.6 (37.8, 71.2)	45.3 (38.1, 73.4)	39.8 (36, 52.7)	0.002
Hospitalized prior to transplant				0.035
Not hospitalized	508 (66.9%)	443 (65.4%)	65 (79.3%)	
Hospitalized	68 (9%)	62 (9.2%)	6 (7.3%)	
In ICU	183 (24.1%)	172 (25.4%)	11 (13.4%)	
Preoperative ventilator	75 (9.9%)	68 (10%)	7 (8.5%)	0.813
Preoperative ECMO	92 (12.1%)	86 (12.7%)	6 (7.3%)	0.218
Days on wait list	32 (11, 89.5)	31 (11, 87)	39 (16, 136.5)	0.204

Variables are either presented as median, interquartile range, or n; percentage when appropriate. BMI: body mass index; ECMO: extracorporeal membrane oxygenation; GFR: glomerular filtration rate; ICU: intensive care unit; LAS: lung allocation score; mPAP: mean pulmonary artery pressure.

Supplemental Table S3. Unmatched donor characteristics of No-EVLP vs. EVLP subgroup who developed PGD3 2018–2023.

Variable	Overall (n = 759)	No-EVLP (n = 677)	EVLP (n = 82)	P-Value
Age	37 (27, 49)	37 (27, 49)	42.5 (27.2, 49)	0.283
Male sex	441 (58.1%)	388 (57.3%)	53 (64.6%)	0.25
Coronary artery disease	59 (7.8%)	54 (8%)	5 (6.1%)	0.703
Smoking history	75 (10.1%)	67 (10.1%)	8 (9.9%)	0.999
Recent cocaine use	161 (21.6%)	150 (22.5%)	11 (13.8%)	0.098
Diabetes	72 (9.6%)	59 (8.8%)	13 (16%)	0.057
Hypertension	199 (26.4%)	173 (25.7%)	26 (31.7%)	0.302
Alcohol abuse	584 (79.6%)	523 (79.8%)	61 (77.2%)	0.689
BMI (kg/m2)	25.6 (22.5, 29.3)	25.4 (22.5, 29)	27.6 (24, 32.2)	0.001
PF ratio	435 (373, 494)	439.6 (376.8, 494.9)	397.5 (333, 472.8)	0.003
Donor cause of death				0.694
Neuro (seizure/CVA)	233 (30.7%)	203 (30%)	30 (36.6%)	
Drug overdose	119 (15.7%)	107 (15.8%)	12 (14.6%)	
Asphyxiation	36 (4.7%)	32 (4.7%)	4 (4.9%)	
Cardiovascular	72 (9.5%)	62 (9.2%)	10 (12.2%)	
Trauma (GSW/stab/blunt)	276 (36.4%)	253 (37.4%)	23 (28%)	
Drowning	1 (0.1%)	1 (0.1%)	0 (0%)	
Other	22 (2.9%)	19 (2.8%)	3 (3.7%)	
DCD	83 (10.9%)	56 (8.3%)	27 (32.9%)	< 0.001

Variables are either presented as median, interquartile range, or n; percentage when appropriate. BMI: body mass index; CAD: coronary artery disease; CVA: cerebral vascular accident; DCD: donation after circulatory death; GSW: gunshot wound; PF ratio: PaO2/FiO2 ratio.

Supplemental Table S4. Unmatched operative and postoperative characteristics of No-EVLP vs. EVLP subgroup who developed PGD3 2018–2023.

Variable	Overall (n = 759)	No-EVLP (n = 677)	EVLP (n = 82)	P-Value
Center volume yearly	19.5 (12.3, 23.3)	19.5 (13.3, 23.3)	16.5 (9.3, 31.1)	0.174
Bilateral lung transplant	645 (85%)	574 (84.8%)	71 (86.6%)	0.675
Distance traveled	164 (74, 344)	157 (71, 296)	329.5 (147.8, 538.2)	< 0.001
Ischemic time	6 (4.8, 7.3)	5.7 (4.6, 6.8)	11.8 (8.6, 15)	< 0.001
Length of stay (days)	32 (18.5, 59)	33 (19, 61)	28.5 (18, 47)	0.222
In-hospital mortality	59 (8.1%)	49 (7.5%)	10 (12.8%)	0.163
Postoperative dialysis	155 (20.4%)	133 (19.6%)	22 (26.8%)	0.168
Postoperative stroke	29 (3.8%)	26 (3.9%)	3 (3.7%)	0.999
Airway dehiscence	15 (2%)	14 (2.1%)	1 (1.2%)	0.915
Postoperative ECMO	170 (22.4%)	136 (20.1%)	34 (41.5%)	< 0.001
Postoperative ventilator				0.226
<2 days	205 (27.2%)	191 (28.4%)	14 (17.5%)	
2–5 days	133 (17.7%)	117 (17.4%)	16 (20%)	
5+ days	401 (53.3%)	353 (52.5%)	48 (60%)	
None	14 (1.9%)	12 (1.8%)	2 (2.5%)	
Acute rejection (hospitalization)				0.118
Yes and treated with immunosuppressant	73 (9.6%)	63 (9.3%)	10 (12.2%)	
Yes and not treated with immunosuppressant	16 (2.1%)	12 (1.8%)	4 (4.9%)	
No	670 (88.3%)	602 (88.9%)	68 (82.9%)	
Treated rejection (1st year)	94 (18.4%)	78 (17%)	16 (30.2%)	0.031
Cause of death				0.673
Graft failure	30 (15.2%)	26 (15.1%)	4 (15.4%)	
Malignancy	4 (2%)	4 (2.3%)	0 (0%)	
Cardio/cerebrovascular	24 (12.1%)	19 (11%)	5 (19.2%)	
Pulmonary	46 (23.2%)	42 (24.4%)	4 (15.4%)	
Infection	57 (28.8%)	48 (27.9%)	9 (34.6%)	
Other	37 (18.7%)	33 (19.2%)	4 (15.4%)	
Perfused by				
Organ procurement organization			2 (2.5%)	
Transplant program			53 (65.4%)	
External perfusion center			26 (32.1%)	
Perfusion time (minutes)			253 (214, 344)	

Variables are either presented as median, interquartile range, or n; percentage when appropriate. ECMO: extracorporeal membrane oxygenation.

Supplemental Table S5. Matched recipient characteristics of No-EVLP vs. EVLP subgroup who developed PGD3 2018–2023.

Variable	Overall (n = 285)	No-EVLP (n = 209)	EVLP (n = 76)	P-Value
Age	62 (54, 67)	62 (54, 68)	60 (54, 65.2)	0.177
Male sex	169 (59.3%)	126 (60.3%)	43 (56.6%)	0.669
Race				0.817
White	209 (73.3%)	153 (73.2%)	56 (73.7%)	
Black	41 (14.4%)	29 (13.9%)	12 (15.8%)	
Other	35 (12.3%)	27 (12.9%)	8 (10.5%)	
BMI (kg/m2)	27.61 +/- 3.91	27.6 +/- 4.1	27.64 +/- 3.37	0.931
Diabetes	43 (15.1%)	33 (15.8%)	10 (13.2%)	0.718
Former smoker >20 pack years	159 (55.8%)	117 (56%)	42 (55.3%)	0.999
GFR (ml/min/1.73 m2)	92.5 (72.3, 116.1)	92.3 (73.1, 115.9)	93.7 (70.6, 117.3)	0.889
Preoperative dialysis	1 (0.35%)	1 (0.49%)	0 (0%)	0.999
mPAP (mm Hg)	25 (20, 32.2)	25 (20, 33)	25 (19, 31)	0.811
Diagnosis				0.996
Cystic fibrosis/immunodeficiency	4 (1.4%)	3 (1.4%)	1 (1.3%)	
Obstructive lung disease	54 (18.9%)	39 (18.7%)	15 (19.7%)	
Pulmonary vascular disease	16 (5.6%)	12 (5.7%)	4 (5.3%)	
Restrictive lung disease	211 (74%)	155 (74.2%)	56 (73.7%)	
LAS	40.7 (36, 51.7)	40.7 (36.2, 51.1)	39.9 (35.9, 52.1)	0.581
Hospitalized prior to transplant				0.741
Not hospitalized	222 (77.9%)	162 (77.5%)	60 (78.9%)	
Hospitalized	19 (6.7%)	13 (6.2%)	6 (7.9%)	
In ICU	44 (15.4%)	34 (16.3%)	10 (13.2%)	
Preoperative ventilator	24 (8.4%)	18 (8.6%)	6 (7.9%)	0.999
Preoperative ECMO	21 (7.4%)	16 (7.7%)	5 (6.6%)	0.959
Days on wait list	36 (12, 96)	34 (11, 92)	37.5 (16, 132.8)	0.625

Variables are either presented as median, interquartile range, or n; percentage when appropriate. BMI: body mass index; ECMO: extracorporeal membrane oxygenation; GFR: glomerular filtration rate; ICU: intensive care unit; LAS: lung allocation score; mPAP: mean pulmonary artery pressure.

Supplemental Table S6. Matched donor characteristics of No-EVLP vs. EVLP subgroup who developed PGD3 2018–2023.

Variable	Overall (n = 324)	No-EVLP (n = 243)	EVLP (n = 81)	P-Value
Age	39 (29, 50)	38 (29, 50)	43 (27.8, 51.2)	0.747
Male sex	180 (63.2%)	133 (63.6%)	47 (61.8%)	0.89
Coronary artery disease	27 (9.5%)	22 (10.5%)	5 (6.6%)	0.437
Smoking history	27 (9.5%)	20 (9.6%)	7 (9.2%)	0.999
Recent cocaine use	37 (13%)	27 (12.9%)	10 (13.2%)	0.999
Diabetes	32 (11.2%)	20 (9.6%)	12 (15.8%)	0.208
Hypertension	87 (30.5%)	63 (30.1%)	24 (31.6%)	0.93
Alcohol abuse	63 (22.1%)	45 (21.5%)	18 (23.7%)	0.821
BMI (kg/m2)	26.5 (23.2, 30.8)	26.4 (23.1, 30.4)	27.4 (24.1, 31.3)	0.314
PF ratio	437.8 (364.2, 492.4)	448 (373, 494)	397.5 (338, 474)	0.008
Donor cause of death				0.995
Neuro (seizure/CVA)	99 (34.7%)	72 (34.4%)	27 (35.5%)	
Drug overdose	39 (13.7%)	29 (13.9%)	10 (13.2%)	
Asphyxiation	12 (4.2%)	8 (3.8%)	4 (5.3%)	
Cardiovascular	37 (13%)	27 (12.9%)	10 (13.2%)	
Trauma (GSW/stab/blunt)	87 (30.5%)	65 (31.1%)	22 (28.9%)	
Other	11 (3.9%)	8 (3.8%)	3 (3.9%)	
DCD	56 (19.6%)	32 (15.3%)	24 (31.6%)	0.004

Variables are either presented as median, interquartile range, or n; percentage when appropriate. BMI: body mass index; CAD: coronary artery disease; CVA: cerebral vascular accident; DCD: donation after circulatory death; GSW: gunshot wound; PF ratio: PaO2/FiO2 ratio.

SUPPLEMENTAL FIGURES

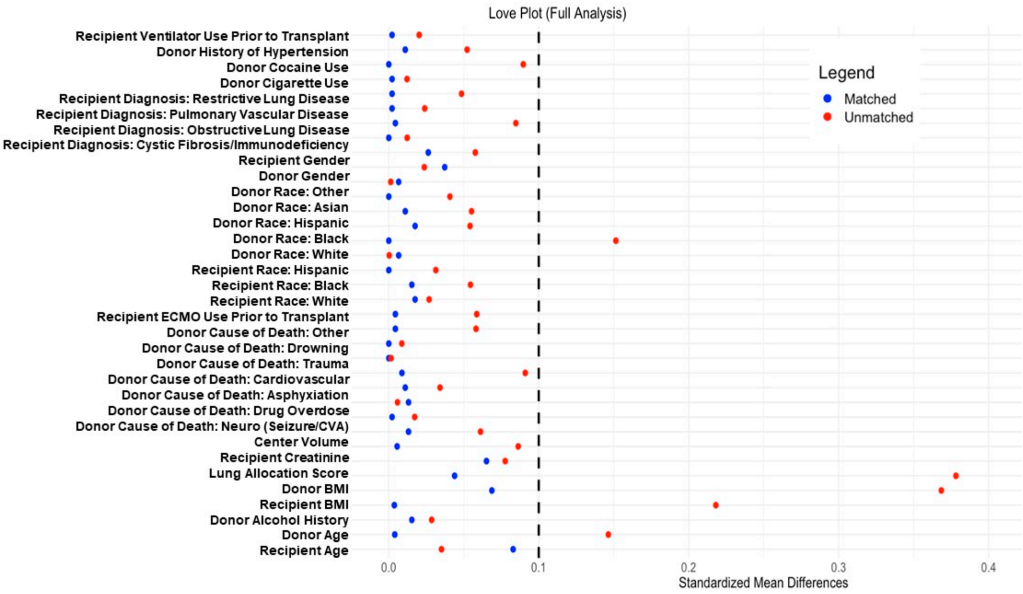

Supplemental Figure 1

Supplemental Figure S1: Love plot of matched and unmatched standard mean deviations for No-EVLP vs. EVLP PGD3 subgroup.

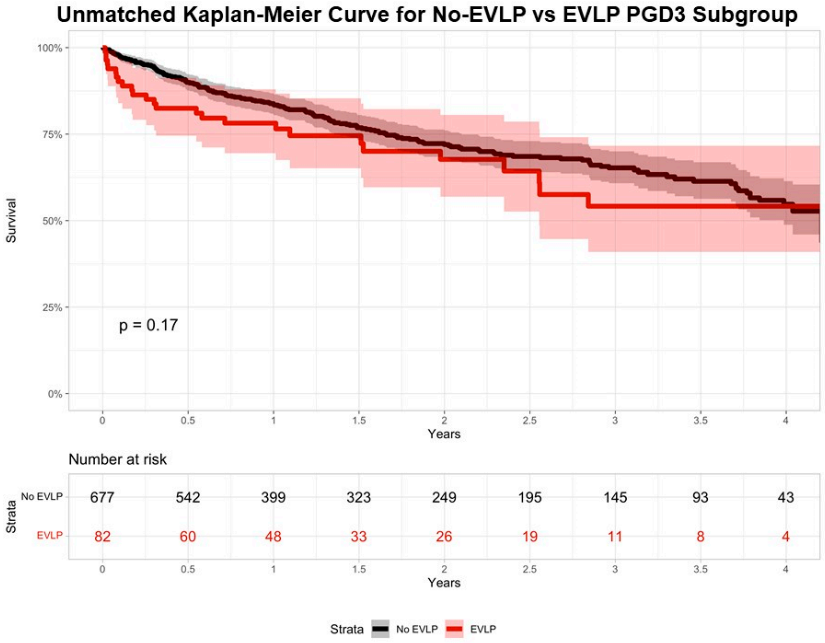

Supplemental Figure 2

Supplemental Figure S2: Unmatched Kaplan–Meier curve of the recipients with PGD3 who underwent No-EVLP or EVLP. Kaplan–Meier survival estimates are plotted; 95% confidence intervals are depicted with shading.
